# A case of intoxication with tea made from *Digitalis purpurea*

**DOI:** 10.21542/gcsp.2021.2

**Published:** 2021-04-30

**Authors:** Anouk Lehmann, Selina Späni, Annette Harings-Kaim, Cecilia Probst, Andreas Christ, Anne B. Leuppi-Taegtmeyer

**Affiliations:** 1 Medical University Clinic, Cantonal Hospital Baselland, Liestal, Switzerland; 2 Hospital Pharmacy, Cantonal Hospital Baselland, Liestal, Switzerland; 3 Regional Pharmacovigilance Centre, Department of Clinical Pharmacology & Toxicology, University Hospital Basel and University of Basel, Basel, Switzerland; 4 Department of Anaesthesia, Cantonal Hospital Baselland, Liestal, Switzerland

## Abstract

We present the case of a 34-year-old woman with recurrent depressive disorder who ingested purple foxglove with suicidal intent. She bought a foxglove plant *(Digitalis purpurea)* over the internet and used all of its leaves to make a tea that she then drank over a period of a few hours. Seventeen hours later, she developed abdominal pain, emesis and bradycardia and was admitted via the emergency department to the intensive care unit for further treatment and monitoring. The plasma digoxin concentration measured 3.53 nmol/l (therapeutic reference range 0.77–1.50 nmol/l) 21 hours after ingestion of the tea. She remained heamodynamically and neurologically stable, was treated with antiemetics and simple analgesia and did not require digoxin-specific antibodies. Despite normal renal function, her plasma digoxin half-life was prolonged (estimated 76 h), reflecting the long half-life of the parent compound digitoxin which is the main cardiac glycoside in *Digitalis purpurea*. She was transferred to psychiatric care 48 h after admission. In this report, we compare this case to other similar cases, which to date have only been rarely reported in the literature.

## Background

Foxglove (Digitalis sp.) is one of a number of medicinal plants containing cardenolides which belong to a group of chemicals called cardiac glycosides. One of these cardiac glycosides - digoxin - was first described as a therapy for heart failure in 1785^1^ and is still widely prescribed today for patients with atrial fibrillation and heart failure or left ventricular systolic dysfunction.^2^

Due to its low therapeutic index, overdoses occur frequently and can be life-threatening. Intoxication manifests itself in symptoms such as nausea, vomiting, dizziness, visual disturbance, alterations of consciousness and bradycardia with AV block. In rare cases, life-threatening tachyarrhythmias, hyperkalaemia, convulsions and coma occur. Cardiac arrest as a result of asystole and ventricular fibrillation can be fatal.^3^

Due to their bitter taste, accidental poisoning with plants containing cardiac glycosides is rare. We present a case where a digitalis extract was made and ingested with suicidal intention. A similar case from the same region in Switzerland was published in the medical literature a year previously.^4^ We therefore reported this current case to the national drug authority (Swissmedic) who have a responsibility in safety monitoring, not only of licensed drugs.

## Case presentation

**Figure 1.**
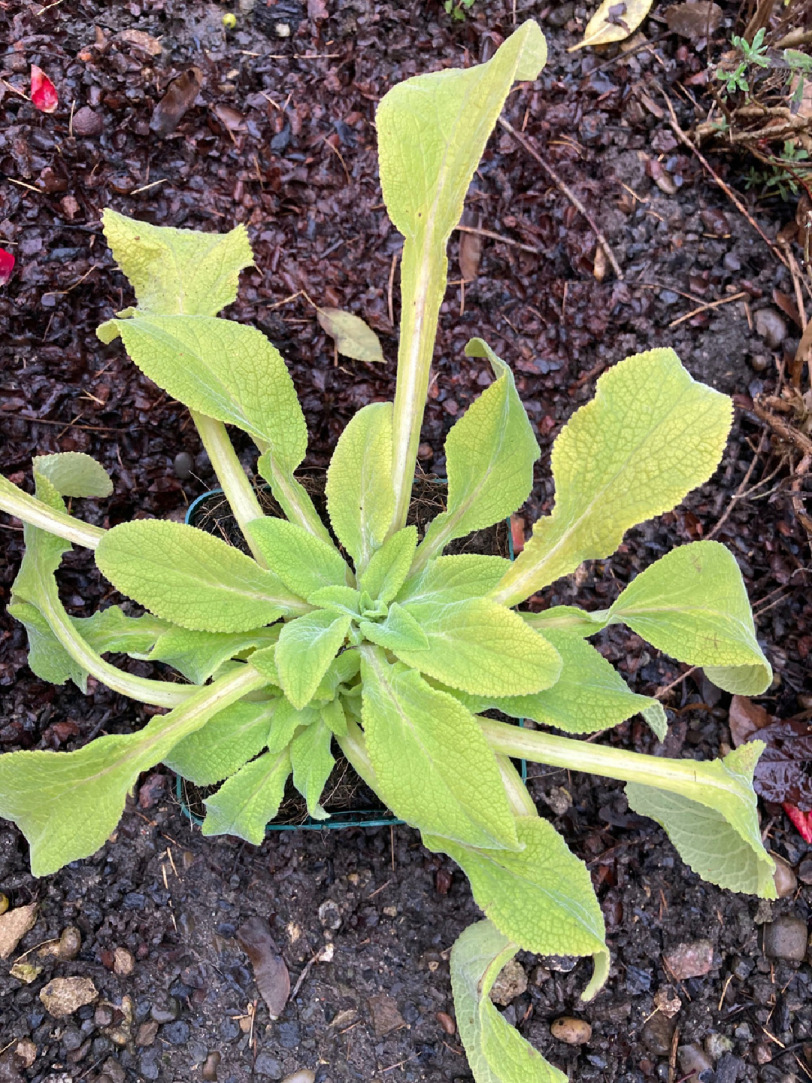
*Digitalis purpurea* showing winter foliage. (Photo credit: A. Leuppi-Taegtmeyer).

We present the case of a 34-year-old woman with recurrent depressive disorder and previous attempted suicide who presented to the emergency services after ingesting purple foxglove. She had ordered a purple foxglove plant via the internet and then used it to brew a tea. As it was autumn, we presume the plant was not in flower and so only had leaves (Figure 1). A few hours after drinking this tea, she vomited several times. The following morning (approximately 17 hours after ingesting the tea) she experienced severe abdominal and thoracic pain and called an ambulance. Her regular medication was escitalopram 20 mg once daily.

On arrival in the emergency room the patient was tearful and looked unwell but was afebrile, alert and orientated. She denied taking escitalopram in a dose greater than the one prescribed. On physical examination she was bradycardic with a regular pulse of 58 bpm and a blood pressure of 129/73 mmHg. Her oxygen saturation breathing room air was 98%. Neurological examination including cranial nerves and pupil size was normal. Cardiac and lung auscultation were normal and she had neither peripheral edema nor an elevated jugular venous pressure. The abdomen was soft with diffuse epigastric tenderness and hyperactive bowel sounds.

Laboratory results at hospital admission showed a normal potassium concentration (4.3 mmol/l [reference range 3.5–5.1 mmol/l]) as well as a normal calcium concentration (2.56 mmol/l [reference range 2.10–2.60 mmol/l]). A plasma digoxin concentration measured four hours after presentation (21 hours after ingestion of the tea) was 3.53 nmol/l [reference range 0.77–1.50 nmol/l]). In accordance with the patient’s history and clinical findings, a plasma escitalopram concentration was not determined.

The patient was admitted to the intensive care unit for further treatment and monitoring. There, she was treated symptomatically with intravenous ondansetron and oral paracetamol. She remained hemodynamically compensated at all times, and, other than bradycardia (lowest heart rate 48 bpm), she experienced no arrhythmias or alteration in mental state. Her clinical and laboratory findings did not fulfill the criteria for antidote treatment with digoxin-specific antibody fragments at any point. She could be discharged to a psychiatric unit 48 hours after admission.

According to the criteria of the World Health Organization (WHO) for assessing causality, the regional pharmacovigilance center classified the causal relationship between the patient’s clinical symptoms and ingestion of digitalis purpurea tea as “certain”.^5^

## Discussion

**Figure 2.**
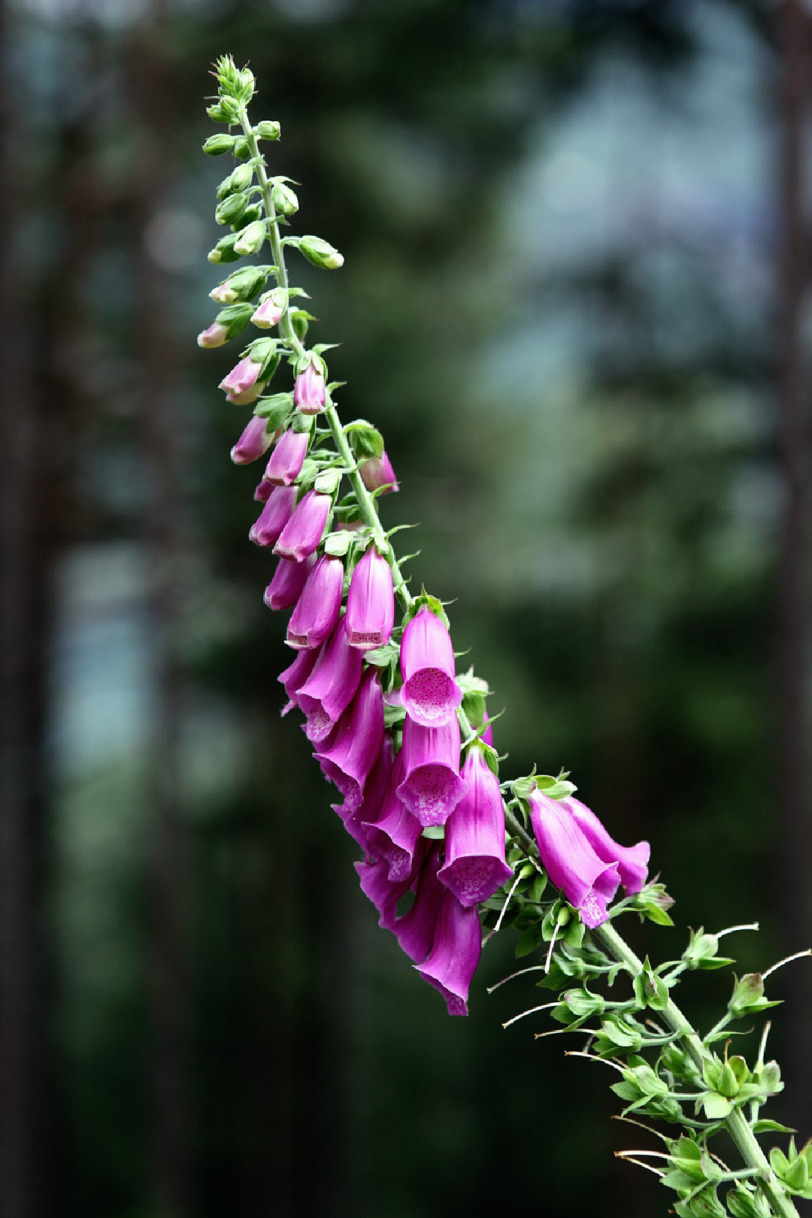
*Digitalis purpurea* in full bloom showing its characteristic purple flowers.

Cardiac glycosides are found in all parts of the purple foxglove plant, *Digitalis purpurea* (Figure 2). The main pharmaceutical ingredient is digitoxin, which has been implicated in other cases of self-harm reported in the literature.^4,6–10^

Signs of toxicity are the same as for other cardiac glycosides (such as digoxin) and include cardiac arrhythmia, gastrointestinal and central nervous symptoms. Manifestations of life-threatening or fatal toxicity include rapidly evolving and changing cardiac arrhythmias, for example progressive bradyarrhythmias, heart block and ventricular arrhythmias, and hyperkalemia.^3,6,9^

Within one or two hours after ingestion of a potentially life-threatening dose of a cardiac glycoside, activated charcoal and colestyramine may be beneficial to prevent absorption or entero-hepatic recirculation.^11^ A good response to repeated doses of activated charcoal for 24 hours after presentation was shown in a recent case of intoxication with Digitalis purpurea.^9^

If life-threatening arrhythmia, evidence of end-organ dysfunction, hyperkaliemia (serum potassium >5–5.5 mmol/L) are present, or digoxin levels exceed 19.2 nmol/L (15 ng/mL) at any time, or 12.8 nmol/l (10 ng/mL) six hours after ingestion, treatment with the antidote digoxin-specific antibody fragments (Digifab^®^ or Digibind^®^) should be initiated.^3^ These fragments bind the free cardiac glycosides in the extracellular space to form inactive glycoside-immunoglobulin complexes which are then renally excreted.

The dose of digoxin-specific antibody fragments is determined either according to the ingested amount of digoxin or the plasma digoxin concentration. When these are unknown, 400–500 mg is given and the dose repeated according to clinical response. As the serum digoxin concentration does not necessarily correlate with toxicity, hyperkalemia is an important marker and prognostic indicator for acute toxicity. Repeated potassium measurements showed values in the normal range and other criteria for antidote treatment were not met in the case we presented here.

The role of digoxin-specific antibody fragments in the treatment of poisoning with foxglove plant extracts was reported in five patients thirty years ago, all of whom responded to therapy.^12^ Specific dosing guidelines are, however, not available. One case reported transient response in terms of reversal of heart block, amelioration of nausea and improved mental status but these effects were not sustained and not associated with accelerated elimination of digitoxin.^8^ The authors suspected that the dose of digoxin-specific antibody fragments – although 1.2 g were administered in total - was insufficient to bind all the extracellular cardiac glycosides.

A recent case also reported transient ECG-responses after repeated digoxin-specific antibody fragment administration summing 760 mg during the 21 hours following ingestion of 20 foxglove leaves.^9^ In a further case, administration of 380 mg of digoxin-specific antibody fragments had no clinical effect.^6^ These observations likely reflect the 30–100 times lower affinity of digoxin-specific antibody fragments to digitoxin compared to digoxin.^12^ Taken together, these data suggest that in cases where digoxin-specific antibody fragment treatment is indicated after poisoning with Digitalis purpurea, large amounts (perhaps even over a number of days) are likely to be required for a favorable response.

Digitoxin is available as a prescription drug in Germany and Austria, but not in Switzerland or other countries. While the more commonly used cardiac glycoside digoxin is not protein-bound to a relevant extent and excreted through the kidneys, digitoxin is highly protein-bound and metabolized by the liver. About 2% of the total digitoxin is hydroxylated to digoxin.^13^ The metabolites are excreted via the bile and are reabsorbed to some extent (entero-hepatic cycling). This results in a half-life of 7 days (compared to 1.5 days for digoxin in patients with normal renal function).^13^

Because digitoxin is not a licensed drug in Switzerland, unlike in Germany, laboratories do not have validated assays for determining digitoxin concentrations. After hospital admission, the plasma digoxin concentration was measured instead to confirm the diagnosis and estimate the toxicity of the digitalis. The electrochemical luminescence immunoassay used in the clinic, does not detect digitoxin as the primary substance. However, the assay manufacturers report significant cross-reactivity for digitoxin.^14^

A digoxin level of 3.53 nmol/l was measured on the day of hospital admission. This value, as well as the value on the day of hospital discharge (2.34 nmol/l), were above the reference range of 0.77–1.50 nmol/l. A half-life of approximately 76 hours was calculated, which exceeds that expected in patients with normal renal function (40 hours), most likely due to the continued generation of digoxin from digitoxin that has a longer half-life. This is in keeping with the observation of a strong correlation between plasma digitoxin and digoxin concentrations in 59 patients taking digitoxin.^15^ Based on this we estimated a maximum digoxin level of around 4 nmol/l 6 hours after drinking the digitalis tea.

Despite confirmed intoxication with a cardiac glycoside, which led to high measured plasma digoxin concentrations, our patient‘s symptoms were not life threatening and she did not require treatment with digoxin-specific antibody fragments. Similar cases of large ingestions of digitoxin which were subsequently relatively well-tolerated have been described in young people who ingested foxglove leaves or extract.^4,7,10,16^ This may be due to the acute nature of the exposure and good hepatic and renal function promoting both high protein-binding and elimination and absence of interacting concomitant medication. Furthermore, younger people are able to tolerate cardiac depression better than older people.

In contrast, a case of digitoxin poisoning after ingestion of a foxglove plant by a 64-year-old man was fatal;^6^ and the cases of a 67-year-old woman and her 66-year-old husband who accidentally consumed foxglove leaves were severe, protracted and required repeated doses of digoxin-specific antibody fragments.^17^ The recent case of a 19-year-old woman who ingested 20 foxglove leaves, however, was associated with relevant hyperkalaemia and bradycardia.^9^ The patient required prolonged treatment, possibly due to impaired gastric emptying causing continued absorption of cardiac glycosides from the plant material. The dose of ingested cardiac glycoside clearly plays a central role and this is not known in any of the cases, so comparisons and speculation about prognostic factors are difficult to make.

In conclusion, we have described an illustrative case of digitoxin intoxication in a real-life setting and have compared it to other cases, which to date have only been rarely reported in the literature. We hope the experience gained will help in the management of future, similar cases.

## Ethics statement

The patient gave their written informed consent for the publication of this case in accordance with the Declaration of Helsinki.
